# Effect of Custom-Made Foot Orthotics on Multi-Segment Foot Kinematics and Kinetics in Individuals with Structural Hallux Limitus

**DOI:** 10.3390/s24196430

**Published:** 2024-10-04

**Authors:** Magdalena Martinez-Rico, Gabriel Gijon-Nogueron, Ana Belen Ortega-Avila, Luis E. Roche-Seruendo, Ana Climent-Pedrosa, Enrique Sanchis-Sales, Kevin Deschamps

**Affiliations:** 1 Department of Nursing and Podiatry, Faculty of Health Sciences, University of Malaga, 29016 Malaga, Spain; magdalenamr96@uma.es (M.M.-R.); anaortavi@uma.es (A.B.O.-A.); 2 KU Leuven, Musculoskeletal Rehabilitation Research Group, Department of Rehabilitation Sciences, Campus Brugge, Spoorwegstraat 12, 8200 Bruges, Belgium; kevin.deschamps@kuleuven.be; 3 IBIMA Plataforma BIONAD, 29590 Malaga, Spain; 4 Facultad de Ciencias de la Salud, Universidad San Jorge, 50830 Zaragoza, Spain; leroche@usj.es (L.E.R.-S.) amcliment@usj.es (A.C.-P.); 5 Facultad de Enfermería y Podología, Universidad de Valencia, C/Jaume Roig s/n, 46010 Valencia, Spain; ensansa@uv.es; 6 Division of Podiatry, Haute Ecole Leonard De Vinci—Institut D’Enseignement Supérieur Parnasse Deux-Alice, 1200 Brussels, Belgium

**Keywords:** kinematics, kinetics, foot orthosis, structural hallux limitus

## Abstract

The first metatarsophalangeal joint (MTPJ) and the first ray are crucial in walking, particularly during propulsion. Limitation in this joint’s sagittal plane motion, known as hallux limitus, can cause compensatory movements in other joints. Some studies assessed the impact of various foot orthoses designs on the foot biomechanics; however, a comprehensive understanding is lacking. This study compared the effects of two custom-made foot orthoses (CFOs) on the foot joint kinematics and kinetics in patients with structural hallux limitus (SHL). In this quasi-experimental study, 24 patients with hallux limitus were assessed in three conditions: (i) barefoot, (ii) shod with a cut-out custom foot orthosis (cut-out CFO), and (iii) shod with an anterior forefoot-stabilized element custom foot orthosis (AFSE CFO), fitted into a minimalist neoprene shoe. Multi-segment foot kinematics and kinetics were assessed during the stance phase of the gait. A decrease in ankle and midfoot inversion, as well as in ankle plantarflexion, was found in both orthotic conditions. Regarding the first MTPJ, a greater dorsiflexion was observed with the patient being barefoot compared to both of the conditions under study. From the current finding, it should be concluded that neither of the custom foot orthoses produced the predefined functional effects.

## 1. Introduction

The first metatarsophalangeal joint (first MTPJ) and the first ray play a pivotal role in the biomechanics of walking, particularly during propulsion [1]. The dorsiflexion of the first MTPJ is a vital component during this phase because it stabilizes the foot, activates the windlass mechanism, and transforms the foot into a rigid lever for propulsion [2,3]. This dorsiflexion is crucial as it allows for efficient load transfer and energy conservation during the gait.

Contrarily, a limitation in this sagittal plane motion can trigger compensatory movements in other joints, such as increased ankle dorsiflexion during midstance and reduced plantarflexion during propulsion. This compensatory mechanism can alter the natural gait pattern, leading to inefficient movement and increased energy expenditure. More proximally in the lower limb kinetic chain, these compensations may lead to increased knee flexion, decreased hip extension [1,2], and potentially chronic postural alteration, like lumbar straightening [4].

Hallux limitus (HL), characterized by the reduced mobility of the first MTPJ in the sagittal plane [5,6], is a prevalent condition affecting approximately 35–60% of individuals around the age of 50 [7,8]. It stands as the second most common cause of first ray involvement [9,10].

Typically, two main categories of hallux limitus (HL) are distinguished as follows: structural HL (SHL) and functional HL (FHL). SHL involves a limitation of the dorsiflexion range of motion in both weight-bearing and non-weight-bearing conditions, whereas FHL is characterized by a restriction in dorsiflexion solely during weight-bearing activities. Some researchers propose that FHL serves as a precursor to SHL [7,8,11]. Understanding the progression from FHL to SHL is essential for early intervention and prevention strategies.

The cause of HL is intricate [12,13], and its etiopathogenesis can range from an impairment of the soft tissues or an insufficiency of the peroneus longus muscle to alterations in the length of the first metatarsal, changes in the shape of its head, or even osteoarthritis [14]. In cases of FHL, it is thought that excessive pronatory forces across the rearfoot and midfoot joints drive the underlying pathomechanics [8]. Genetic predispositions, biomechanical abnormalities, and systemic conditions such as diabetes mellitus and rheumatoid arthritis have also been implicated in the development and progression of HL, adding to its complexity and exacerbating joint degeneration. Symptoms vary depending on the severity of the condition [13,14]. The most common symptom is pain in the first MTPJ. The patients sometimes experience burning pain, with irritation of the dorsal digital branch of the medial dorsal cutaneous nerve and paraesthesia. At the skin level, a tyloma can occur. The symptoms are aggravated by walking barefoot, wearing heels, or wearing very flexible sport shoes [15,16].

In the case of SHL there are a wide variety of classification systems, based on pain and radiological findings. Conservative treatment options in HL encompass functional taping [13], exercises, manipulations [17], modifications, or custom-made foot orthoses (CFO) [15,18]. For individuals who have been diagnosed with FHL, a typical treatment approach entails utilizing a foot orthosis that features a medially positioned wedge to control external pronatory moments (a force or torque applied externally that causes or contributes to the inward rotation (pronation) of the foot or lower limb. This moment acts around a specific axis, typically leading to increased pronation during the gait or stance, which can influence the foot’s alignment and overall biomechanics) [17,19,20]. However, this geometric feature (This term refers to the physical dimensions, shapes, and structural characteristics of orthotic devices. These features include aspects such as the curvature, thickness, arch height, and overall contour.) may not suffice for patients with SHL [19]. Another treatment outlined in the literature is the utilization of a cut-out CFO. It consists of an insole, into which a fenestration is made at the level of the head of the first metatarsal. The design varies in two different ways: some authors perform only a fenestration of the head of the first metatarsal, while others, in addition to this fenestration, add an extension at the level of the hallux (known as dynamic wedge extension) to place the proximal phalanx in an even more dorsiflexed position with respect to the first metatarsal [21,22,23,24,25]. A limited number of studies have investigated the efficacy of this foot orthosis design and have reported an increased plantarflexion of the first ray [3,24], enabling the proximal phalanx to dorsiflex over the metatarsal head. The latter is taught to reduce joint compression, particularly during the propulsion phase [24,26]. Nonetheless, there is a lack of research examining the biomechanical alterations across different foot segments. Similarly, only one study [21] has analyzed these changes throughout the various phases of the gait cycle. The limited body of evidence underscores the need for more comprehensive research to validate these treatments modalities.

In conjunction with the cut-out CFO, additional geometric features have been implemented. These may involve incorporating a rocker element at the forefoot, such as the anterior forefoot stabilizer element (AFSE) CFO. The inclusion of this element aims to enhance the range of motion of the first MTPJ, optimize load transfer between the first MTPJ and the hallux, and promote propulsion. Nevertheless, the effectiveness of these geometric features has yet to be thoroughly investigated.

Hence, while certain investigations have explored the influence of various foot orthoses designs on plantar pressure distribution and foot joint kinematics, a comprehensive understanding is not yet available. Therefore, it is crucial to highlight the present scarcity of research on the effects of foot orthoses on joint kinetics, particularly concerning joint moments and joint power. Given the biomechanical challenges presented by hallux limitus (HL), the design and effectiveness of foot orthotics require detailed rationale. Orthoses aimed at managing HL must specifically address the impaired windlass mechanism and the associated reduction in first MTPJ mobility by enhancing first ray stabilization; foot orthotics can potentially restore proper function and alleviate symptoms. This interaction underscores the need for in-depth studies focused on how orthoses impact joint kinetics in HL, providing critical insights that could lead to optimized orthotic designs and improved patient outcomes. Regarding the biomechanical significance of the first MTPJ and its interaction with other foot and lower limb joints, detailed investigation into these kinetics’ aspect is warranted. Such research could provide insights into optimizing foot orthoses designs to improve patients’ outcomes.

Therefore, the objective of this study was to compare the effect of two different types of CFO on the multi-segment foot joint kinematics and kinetics of patients with SHL. This study aims to fill the existing gaps in the literature and contribute to evidence-based practices in the management of SHL.

## 2. Materials and Methods

### 2.1. Design

The study employed a quasi-experimental design incorporating a repeated-measures approach with a single study population. Each participant was evaluated through three different conditions: (1) barefoot, (2) shod with a cut-out CFO, and (3) shod with an AFSE CFO.

### 2.2. Participants

Patient recruitment took place at the University of San Jorge, a private clinic, and at various sports clubs across the city between October 2021 and December 2022. A convenience sample of 24 participants (11 females 13 males) with SHL participated in the study, with a mean age of 34.2 years (±7.9) and a mean BMI of 23.9 kg/m^2^ (±2.6) (Table 1). The inclusion criteria comprised the following: (1) patients aged between 18 and 65 years; (2) individuals diagnosed with SHL (assessed using a goniometer in a non-weight bearing state, with a first MTPJ range of motion less than 60 degrees); (3) a positive result on the Jacks test [26]; (4) a positive lunge test [27]; and (5) a pronated foot posture index (FPI) (greater than 6). The exclusion criteria encompassed patients with neurological, systemic, or orthopedic conditions, those who had experienced foot or lower limb trauma before the study, and individuals unable to walk without assistance.

This study adhered to the principles outlined in the Declaration of Helsinki regarding the ethical standards for medical research involving human subjects. Approval was obtained from the Ethics Committee of the University of Malaga (CEUMA-12-2021-H), and all participants provided signed informed consent.

### 2.3. Intervention

**Figure 1:**
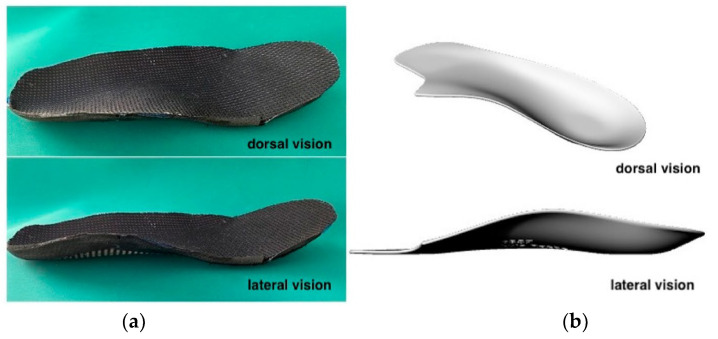
(**a**) AFSE CFO. (**b**) Cut-out CFO.

Each participant received two distinct CFOs. The first CFO was manufactured using a direct molding technique, employing a vacuum-forming machine. This process involved integrating an AFSE CFO with the subtalar joint, positioned neutrally. The resulting orthosis featured a rocker-shaped anterior portion extending from the metatarsal areas to the toes. Polyester resin was utilized as the primary material for the orthosis, with a combination of 1.2 mm podiaflex and 1.2 mm podiaflux (podiatech) for the rearfoot and midfoot, and 0.8 mm podiaflex for the forefoot. The shell consisted of a 30 Shore A and 148 kg/m³ density polyethylene-ethylene-vinyl-alcohol (PE-EVA) (Figure 1a). The choice of PE-EVA with a 30 Shore A hardness and a density of 148 kg/m^3^ is crucial in cases of hallux limitus because it provides the necessary balance between flexibility and resistance. The material’s moderate hardness allows for sufficient load distribution across the foot, while the density ensures the structure is firm enough to limit the excessive dorsiflexion of the hallux during the gait. Specifically, in cases of hallux limitus, it is essential to control dorsiflexion at the first metatarsophalangeal joint (MTPJ) to prevent pain and potential worsening of the condition. The 30 Shore A hardness provides enough compliance to accommodate the foot’s movements without excessively restricting motion, yet it prevents the big toe from dorsiflexing beyond its pain threshold. Furthermore, the density of 148 kg/m^3^ ensures that the material maintains its structural integrity under the forces experienced during ambulation, particularly in the propulsive phase, where control over hallux movement is critical to avoid further joint damage [28]. This combination of material properties was chosen to strike an optimal balance between providing pain relief and promoting a functional gait in individuals with SHL.

The second orthosis, termed the cut-out CFO, was manufactured using 3D printing technology via HP multijet fusion. The anatomic volumetric foot model for the AFSE CFO was digitized by scanning both the feet of each participant using a Structure Sensor (Occipital Incs, Boulder, CO, USA), along with acquisition software developed by TechMed3D V3 (3D Size Me app Lévis, QC, Canada). This setup comprised three cameras, an onboard inertial measurement system (IMU), and an NU3000 processor, offering a depth resolution of 1280 × 960. The orthosis design was created using Rhino 3D and Rhino 6, incorporating a full contact medial arch and a cut-out at the first metatarsal head, along with a 1 mm EVA extension under the hallux (as depicted in Figure 1b). Polyamide 12 (PA 12) was employed for the shell, while a combination of 30 Shore A and 148 kg/cm^3^ density PE-EVA was utilized for the stabilizing forefoot element and the top cover.

All the participants wore minimalist shoes (SAGUARO neoprene shoes, Fujian, China). These minimalist shoes have a lightweight, durable, non-slip sole, breathable insoles for impact absorption, and a 91% polyester, 9% spandex upper, providing soft, sock-like comfort without laces (Amazon Standard Identification Number B0DCNSN23Z)), chosen for their ability to accommodate various foot orthotics and assumed to have a minimal mechanical impact on foot function [29]. The participants were allowed to test the minimalist footwear at home for several days to become familiar with its use.

### 2.4. The Gait Assessment

**Figure 2:**
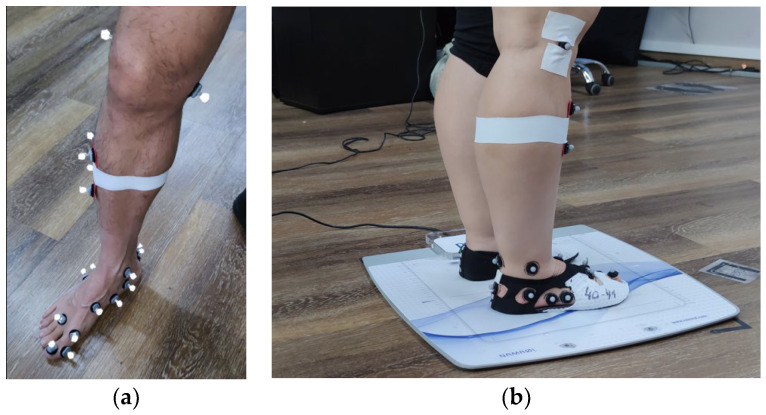
(**a**) Reflective markers following Bruening model without shoe. (**b**) Reflective markers with minimalist shoe.

The gait analysis involved the simultaneous recording of joint kinematics and the normal component of the ground reaction force using a pressure platform. The study employed an adaptation of the model proposed by Bruening et al. [30,31] to capture the kinematics of the ankle, midfoot, and first metatarsophalangeal joints. This model divides the foot into three segments: the rearfoot, midfoot, and hallux. The intersegment angles include the ankle joint (connecting the rearfoot segment to the leg segment), the midfoot joint (MT) (connecting the forefoot segment to the rearfoot segment), and the metatarsophalangeal joint (MPJ) (connecting the hallux segment to the forefoot segment). The model uses 20 reflective markers attached to anatomical reference points on the leg and foot (see Figure 2a). To ensure the optimal marker placement, the shoes were trimmed so that each marker could be precisely aligned with the anatomical landmarks, rather than being placed on the fabric of the shoe, thus mitigating any potential movement caused by the material (see Figure 2b). The three-dimensional (3D) motion of the markers was measured using an eight-camera infrared motion analysis system (Vero, Vicon^®^ Motion Systems Ltd., Oxford, UK), operating at a 100 Hz sampling rate.

The participants were asked to walk through the study laboratory at a comfortable, self-selected speed. A pressure platform (0.40 × 0.40 m, 4 mm thickness, and 0.15 mm sensor thickness; Podoprint, Namrol Group, Barcelona, Spain) was placed at the center of the image acquisition area, where visibility was optimal. The 3-step protocol was used for data collection [32]. Before starting the data collection, the participants were given 10 min to familiarize themselves with the walking conditions. During this familiarization period, they were instructed to maintain a constant speed and avoid deviating from their intended path. Their gait was closely observed to ensure that their gait pattern remained consistent across the different study conditions.

To minimize any potential carryover effects, a rest period of 10–15 min was provided between each change in study conditions. During these breaks, the placement of all the reflective markers was rechecked to ensure accuracy. Each participant was assessed under three different conditions on the same day, with the order of conditions randomized. A total of ten walking trials were recorded for each participant. From these, the five trials with the highest visibility and the fewest instances of marker loss (i.e., minimal gaps in the data) were selected for further analyses.

### 2.5. Data Processing

The 3D coordinates of the markers at each moment were utilized to determine segment position and orientation. Subsequently, the joint angles were calculated at each moment from the upright standing static reference posture, with the participant in a relaxed position. This reference posture was recorded for each subject at the beginning of the experiment. The joint angles were derived using a Cardan rotation sequence between the distal and proximal segments, encompassing dorsiflexion/plantarflexion (DF/PF), abduction/adduction (AB/AD), and inversion/eversion (IN/EV). To ensure data accuracy, all the kinematic data underwent low-pass filtering using a 4th-order Butterworth filter with a cut-off frequency of 10 Hz.

The pressure platform (Namrol Group, Barcelona, Spain) was integrated with the infrared camera system to capture contact pressure distribution at a sampling rate of 100 Hz.

The pressure data needed segmentation to ensure that each pressure sensor’s readings corresponded to the correct foot segment. This was accomplished by matching the coordinates of the pressure sensors with the anteroposterior positions of the ankle, MT, and MPJ joint centers. Subsequently, the normal components of the ground reaction forces and the centers of pressure were computed for each frame on each foot segment using the segmented pressure data.

The 3D joint moments were determined by calculating the cross-product of the ground reaction forces acting on distal segments and the 3D distances between the pressure centers and the joint rotation centers, as outlined by Bruening et al. [31].

The influence of foot weight, along with foot angular velocity and linear and angular accelerations, was disregarded in the joint moments’ calculations. The joint moments were expressed relative to the orientation of the local segment frame of the proximal segment.

The joint flexion moments were presented as a percentage of the stance phase during the gait cycle and, in accordance with previous publications [32], amplitudes were normalized to body weight. All the kinetic data were processed with a low-pass filter using a fourth-order Butterworth filter set at a cutoff frequency of 50 Hz.

### 2.6. Sample Size

For the three conditions, the required sample size was calculated using a 95% confidence level and 80% power. This power level ensures a reasonable probability of detecting a significant difference if it truly exists. The effect size was determined based on the smallest observed difference between the means of the conditions, using the pooled standard deviation. The analysis concluded that 24 participants per group were needed to achieve the study’s objectives with the specified confidence and power levels.

### 2.7. Statistical Analysis

The mean joint 3D rotations and joint moments throughout the stance phase of the gait were compared between the conditions using a one-dimensional, statistical parametric-mapping ANOVA (SPM(F)), followed by post-hoc SPM *t*-tests (SPM(t)). In cases where the SPM(F) clusters exceeded the critical threshold, paired *t*-tests (SPM(t)) were employed to compare the conditions. All the SPM1D analyses were conducted using the open-source SPM1D code (v.M0.1, www.spm1d.org) in MATLAB (R2021a, 8.3.0.532, The MathWorks Inc., Natick, MA, USA). To provide a functional interpretation of the 1D data, the following terminology was adopted: (1) loading response (0–19%), (2) midstance (19–50%), (3) terminal stance (50–83%), and (4) pre-swing (83–100%). Cohen’s d was computed to measure the effect size between the different conditions (barefoot, AFSE CFO, and cut-out CFO) across the various regions of the foot (ankle, midfoot, and the first MTPJ) and axes (sagittal, frontal, and transverse). The effect size was calculated by dividing the difference between the means of the two groups by the pooled standard deviation, with the values interpreted as small (d = 0.2), medium (d = 0.5), or large (d = 0.8) effects. The analysis was conducted across four phases of the stance: loading response (0–19%), midstance (19–50%), terminal stance (50–83%), and pre-swing (83–100%). A *p*-value of less than 0.005 was considered statistically significant.

## 3. Results

Twenty-four patients were included in the study: eleven males and thirteen females. There was no loss of any of the initial participants.

### 3.1. Foot Joint Kinematics

***Ankle***

At the ankle, a 1D SPM repeated measure ANOVA analysis revealed a higher degree of plantarflexion during propulsion in the barefoot condition compared to the AFSE CFO condition (*p* = 0.039) and a more plantarflexed position at the initial contact/loading responds compared to the AFSE CFO (*p* = 0.048) and the cut-out CFO (*p* = 0.036) conditions.

The statistical analysis revealed significant differences in the frontal plane between the barefoot condition and the AFSE CFO condition, from 98% to 100% of the stance phase (*p* = 0.049), as well as between the barefoot condition and the cut-out CFO condition, from 92% to 100% of the stance phase (*p* = 0.038). In both cases, the barefoot condition was associated with a greater degree of ankle inversion during the propulsion phase.

**Figure 3:**
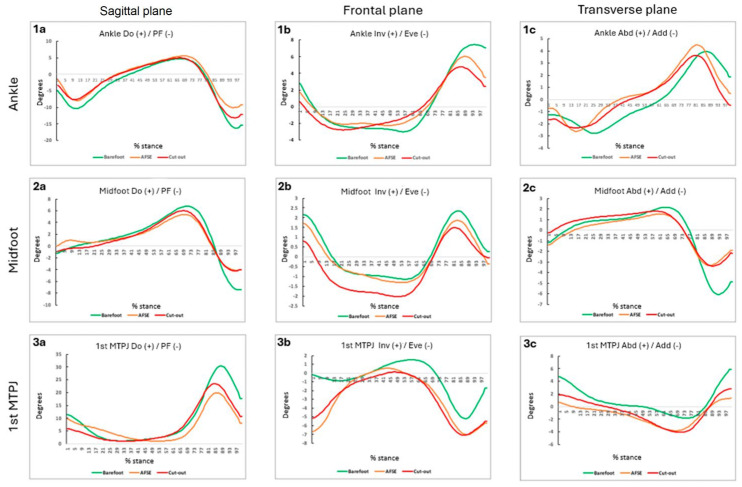
Graphical synthesis about kinematics data. (+) Dorsiflexion (Do), inversion (Inv), abduction (Abd)/(−), plantarflexion (PF), eversion (Eve), and adduction (Add). % stance= percentage of the stance phase. 1 Ankle kinematics, 2 Midfoot kinematics 3 1st Metatarsal phalangeal joint (**a**) sagittal plane; (**b**) frontal plane; (**c**) transverse plane.

As observed in Figure 3(1b), there is a noticeable difference in the position of the graph for the barefoot patient (depicted in green) compared to the patient under the study conditions (depicted in orange and red). In the former case, higher values were reached (positive), reflecting an increase in ankle inversion.

***Midfoot***

The statistical analysis revealed a higher degree of plantarflexion during propulsion in the barefoot condition compared to cut-out CFO condition (*p* = 0.0039) and the AFSE CFO condition (*p* = 0.043).

In Figure 3(2a), it can be observed that the graph corresponding to the barefoot patient (shown in green) is positioned much lower compared to both of the study conditions, particularly at the end of the stance phase, reflecting a greater degree of plantarflexion in the midtarsal joint.

In the frontal plane kinematics, the cut-out CFO condition showed a higher degree of eversion during midstance compared to the barefoot condition from 0% to 25% of the stance phase (*p* = 0.002) and from 42% to 56% of the stance phase (*p* = 0.014). The cut-out CFO condition also exhibited greater eversion compared to the AFSE CFO condition (*p* = 0.001).

In the transverse plane, statistical differences were found between the barefoot and the AFSE CFO (*p* = 0.019) conditions and between the barefoot and the cut-out CFO (*p* = 0.020) conditions in the propulsion phase. In both cases, the barefoot condition was characterized by a higher degree of adduction. A statistical difference was also found between the AFSE CFO and the cut-out CFO conditions in the loading response phase, with a higher degree of abduction in the case of the cut-out CFO condition (*p* = 0.033).

***First metatarsophalangeal joint***

Firstly, on the sagittal plane, the barefoot condition showed a higher degree of dorsiflexion, especially during the propulsion phase, compared to the AFSE CFO (*p* = 0.001) and the cut-out CFO (*p* = 0.005) conditions. In Figure 3(3a), a noticeable difference was observed between the barefoot and the AFSE CFO and the cut-out CFO conditions. 

Furthermore, a statistical difference was observed between the AFSE CFO and the cut-out CFO conditions. The last seems to provide an earlier hallux dorsiflexion compared to the AFSE CFO condition during the terminal stance phase (*p* = 0.005)

With respect to the frontal plane, statistical differences were observed between the barefoot and the AFSE CFO conditions in the loading response (*p* = 0.0037), terminal stance (*p* = 0.030), and propulsion phase (*p* = 0.046). 

The analysis revealed statistical differences between the barefoot and the cut-out CFO conditions in the loading response (*p* = 0.035), terminal stance (*p* = 0.019), and propulsion phase (*p* = 0.041), too.

In the barefoot condition, the hallux is in a more everted position.

Regarding the transverse plane, the 1D SPM repeated measure ANOVA analysis revealed a statistical difference between the barefoot and the AFSE CFO conditions in the loading response (*p* = 0.008), midstance (*p* = 0.001), and propulsion phase (*p* = 0.042) and between the barefoot and the cut-out CFO conditions in the loading response (*p* = 0.022) and terminal stance (*p* = 0.046). In both cases, the barefoot condition was characterized by a higher degree of abduction.

A graphical synthesis about the kinematics data is shown in Figure 3.

### 3.2. Foot Joint Kinetics

***Ankle***

Regarding the sagittal plane, the AFSE CFO and the cut-out CFO conditions showed a higher external dorsiflexion moment during the midstance and the terminal stance compared to the barefoot condition (*p* < 0.001).

With respect to the ankle moment in the frontal plane, a statistical difference was found between the barefoot and the AFSE CFO (*p* = 0.005) conditions and between the barefoot and the cut-out CFO conditions in the propulsive phase (*p* = 0.049). In both cases, the barefoot condition was characterized by a higher external inversion moment.

The significant differences are shown in Table 2.

***Midfoot***

Regarding the frontal plane kinetics, the analysis revealed a statistical difference between the cut-out CFO and the barefoot conditions and between the cut-out CFO and the AFSE-CFO conditions in all the stance phases (*p* < 0.001). The cut-out CFO condition significantly reduced the external eversion moment at the midfoot. (Table 2).

**Figure 4:**
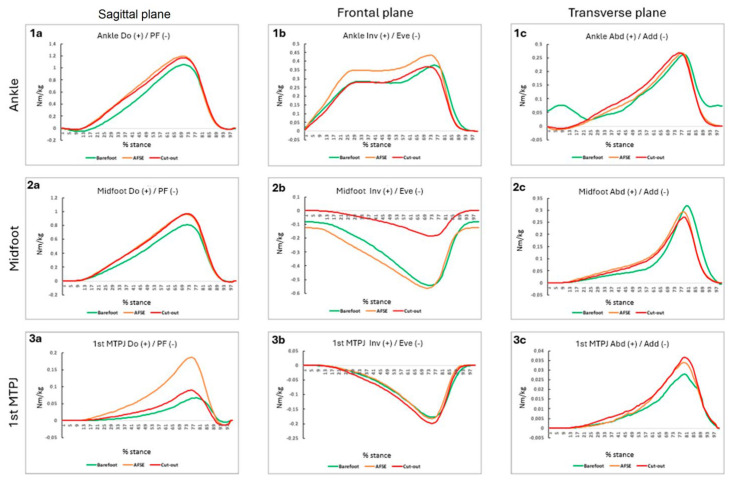
Graphical synthesis about kinetic data. (+) Dorsiflexion (Do), inversion (Inv), abduction (Abd)/(−), plantarflexion (PF), eversion (Eve), and adduction (Add). Nm/kg = newton metro/kilogram. % stance = percentage of the stance phase. 1 Ankle kinetic, 2 Midfoot kinetic 3 1st Metatarsal phalangeal joint (**a**) sagittal plane; (**b**) frontal plane; (**c**) transverse plane.

As shown in Figure 4(2b), the barefoot and the AFSE CFO conditions (depicted in green and orange, respectively) exhibited much lower values compared to the cut-out CFO condition, clearly distinguishing themselves from it.

***First metatarsophalangeal joint***

Statistical differences were found in the sagittal plane, more particularly in the terminal and the propulsion phase.

A higher hallux dorsiflexed moment was observed with the AFSE CFO condition compared to the barefoot and the cut-out CFO (*p* < 0.001) conditions (Table 3). Upon examining Figure 4(3a), it can be observed that the AFSE CFO condition (depicted in orange) reaches significantly higher positive values compared to the other two study conditions. Since positives values correspond to dorsiflexion moments, this indicates an increased dorsiflexion with the AFSE CFO condition.

## 4. Discussion

The results of our study reveal significant changes in the 3D foot joint kinematics and the kinetics of the three joints examined. Contrary to our initial hypothesis, we found a decrease in ankle and midfoot inversion, as well as in ankle plantarflexion, in both of the CFO conditions.

With respect to the ankle joint, the most notable differences were observed during the propulsive phase of the gait. Specifically, there was a decrease in ankle inversion and plantarflexion in both of the CFO conditions. These findings suggest a potential negative impact on the windlass mechanism and on the forefoot rocker function, which is a surprising finding.

We theorize that, particularly, the reduced plantarflexion of the ankle joint during propulsion is caused by an ineffective CFO–shoe coupling during this phase. Indeed, since the current study involved the application of minimalist footwear, we assume that the complete absence of heel drop and the inadequate support base for the orthotics precluded the adequate force transition towards the ground, especially during the third rocker of stance. This reasoning may also explain the higher external dorsiflexion moments during the midstance and the terminal stance that were observed in the current study.

Furthermore, the findings associated with the ankle kinematics and kinetics can be linked to those observed at the MT. The current study highlighted a decreased adduction and plantarflexion of the MT during the propulsion phase. This result indicates less control over the MT with this type of orthotic, which could be related to their design. This suggests a limitation of the forefoot push-off and a trend towards a reduced locking of the midfoot in both of the custom foot orthotics. This diminished control might contribute to a suboptimal energy transfer and suboptimal stability during the gait, emphasizing the importance of midfoot mechanics in overall foot function.

Regarding the first MTPJ, we observed greater dorsiflexion with the patient being barefoot compared to both of the CFO conditions. This contrasts with the findings from other studies [24], which demonstrate that using a cut-out CFO in patients with HL results in an increased angle of declination of the first metatarsal, thereby increasing the dorsiflexion of the first MTPJ. This is the only study we have found that aligns with the objective of ours; however, its methodology differs somewhat, as it studies functional, not structural, HL. The reduced dorsiflexion of the first MTPJ in both of the CFO conditions also provides a plausible explanation for the reduced ankle and midfoot inversion that has been reported here. The first MTPJ plays a crucial role in the resupination of the subtalar joint, since the dorsiflexion of the first MTPJ activates the well-known windlass mechanism created by the plantar aponeurosis on the calcaneus.

The primary objective of this element is to position the first metatarsal in greater plantarflexion and, consequently, the proximal phalanx in more dorsiflexion relative to the first metatarsal, thereby reducing compression in the joint during the propulsion phase [23]. However, few studies have analyzed whether cut-out insoles truly fulfill this function. Welsh et al. [25], who evaluated the effectiveness of cut-out foot orthoses in relieving pain in patients with HL, did not find changes in the dorsiflexion of the first MTPJ or in the correction of rearfoot eversion with the study insoles, although they did achieve pain relief. Other studies [24] suggest that this pain relief may be due to the redistribution of forefoot loads, shifting them to the midfoot during midstance and to the lesser toes. However, it could not be determined that they had a biomechanical functionality. Comparing these articles is difficult because they differ in their methodologies, orthosis designs, and measurement tools.

In addition to this cut-out CFO, there are other treatments that seem to improve the functionality of the first MTPJ [33], such as Morton’s extension. However, various authors state that it is usually used in the more advanced stages of the disease (hallux rigidus) [34]. In our case, we are studying a less advanced stage, and, therefore, we cannot correlate our results with those found by these authors.

The findings from this study have significant clinical implications in the field of biomechanics, particularly concerning the design and prescription of foot orthotics for patients with specific foot pathologies, such as SHL. The reduction in ankle and MT inversion, along with decreased ankle plantar flexion during propulsion, suggests that minimalist footwear combined with certain orthotic designs may not provide sufficient mechanical support or alignment for optimal foot function. Clinicians should take these biomechanical effects into account when recommending orthotic interventions, particularly for patients who need enhanced stability and an efficient force transmission during the gait. Additionally, the reduced dorsiflexion of the first MTPJ points to the need for orthotic designs that effectively engage the windlass mechanism to improve subtalar joint resupination. Future orthotic prescriptions may benefit from incorporating design elements that better support the foot’s natural biomechanics, potentially through the use of customized features that address individuals’ gait abnormalities.

In relation to the optimal properties of orthoses for managing SHL, our study suggests that the ideal orthotic material should balance flexibility and rigidity to provide sufficient dorsiflexion control while allowing some degree of motion at the first MTPJ. The use of materials with moderate hardness, such as 30 Shore A with a density of around 148 kg/m³ (as used in our orthoses), offers the necessary support to limit excessive dorsiflexion and ensure an efficient force transfer during propulsion. This balance is critical in hallux limitus patients, as it prevents pain and further joint degeneration while allowing functional movement. The literature supports that orthotic interventions with materials of similar properties help engage the windlass mechanism, contributing to midfoot stability and enhancing resupination during the gait [19,35]. Furthermore, additional design features, such as forefoot cut-outs or modifications like Morton’s extensions, could further enhance the orthotic’s efficacy in managing SHL by reducing compression at the first MTPJ [21].

From the current findings, it should be concluded that neither of the CFOs produced the predefined functional effects. We propose two main reasons for these results. First, the study population, which consists of patients with SHL, may have difficulty achieving increased mobility due to the structured nature of their pathology. Therefore, they could benefit more from geometric features that aggressively facilitate sagittal plane biomechanics, such as rocker-bottom shoes or shoes with pronounced toe springs, in line with the sagittal plane facilitation theory. Another explanation for the current findings may be found in an inappropriate mechanical matching between the CFO and the minimalist footwear (a poor CFO–shoe coupling). 

The initial decision to use minimalist footwear was made to avoid any external factors or corrections that might be caused by conventional footwear, which typically uses rigid, shock-absorbing materials, etc. The focus was on isolating the pure biomechanical effect that these CFOs can produce. Future studies should include footwear with other plantar and dorsal mechanical properties. 

Investigating the impact of varying heel-to-toe drops, cushioning properties, and the stiffness of the shoe–orthotic interface could provide deeper insights into how different configurations affect the gait mechanics. Additionally, exploring different orthotic materials and designs that better match the mechanical properties of the footwear could enhance the functional outcomes in order to fully appreciate the functional effect of the CFOs. Moreover, it would be beneficial to conduct longitudinal studies to assess the long-term effects of different orthotic interventions on foot kinematics and kinetics to understand how adaptation over time influences the efficacy of orthotic devices.

Despite offering valuable insights, our study had methodological limitations. First, the sample size was small, and future studies would need to repeat the same process using a larger sample. On the other hand, we only studied the direct effect of the CFOs, so we do not know if by studying the CFO in the long term, respecting the process of the adaptation of the patient to the CFO, the results would have been slightly different. Finally, the body of research found was quite scarce, which made it difficult to compare with other authors, making it possible to be less critical when interpreting our results.

It is also recommended to further explore the functional effects of the aforementioned studies using conventional footwear with heel drops and rocker bottoms, since it is reasonable to assume that the external support provided by these shoes would be beneficial for the foot orthotic therapy.

## 5. Conclusions

Significant changes were observed in the 3D foot joint kinematics and kinetics of the three joints examined. However, neither of the CFOs produced the predefined functional effects.

A decrease in ankle and midfoot inversion as well as in ankle plantarflexion were observed in both of the foot orthotic conditions. 

In the first MTPJ, greater dorsiflexion was observed with the patient being barefoot compared to both of the CFO conditions.

Clinicians should consider these biomechanical impacts when recommending orthotic interventions, especially for patients requiring enhanced stability and force transmission during the gait.

## Figures and Tables

**Table 1:** Demographic characteristics.

Demographic Characteristics	Mean and SD
Participants	24 (11 male 13 female)
Age (years)	34.4 ± 8
Height (cm)	169 ± 7.4
Weight (kg)	68.5 ± 9.3
BMI (kg/m^2^)	23.8 ± 2.7
Shoe size	40.5 ± 2.2
Foot posture index	7.6 ± 1.2
Degrees of dorsiflexion (°)	42 ± 7.7

BMI (Body Mass Index); cm (centimeter); kg (kilogram).

**Table 2:** Ankle and midfoot moments. Critical threshold: the specific value or point at which a significant change occurs in the system or variable being measured.

Plane	Comparison	Critical Threshold (t)	Probability Value (*p*)	Percentage of the Stance Phase (%)	Cohen’s d
Ankle sagittal	Barefoot vs. AFSE CFO	3.330	*p* < 0.001	16–66%	0.34
	Barefoot vs. Cut-out CFO	3.267	*p* = 0.005	27–37%	0.19
	AFSE CFO vs. Cut-out CFO	3.744	-	-	
Midfoot sagittal	-	-	-	-	
Ankle frontal	Barefoot vs. AFSE CFO	3.766	*p* = 0.005	87–91%	0.24
	Barefoot vs. Cut-out CFO	3.693	*p* = 0.049	0–2	0.57
	Barefoot vs. Cut-out CFO	3.693	*p* < 0.001	88–93%	0.33
	AFSE CFO vs. Cut-out CFO	3.745	-	-	
Midfoot frontal	Barefoot vs. AFSE CFO	2.914	-	-	
	Barefoot vs. Cut-out CFO	2.844	*p* < 0.001	0–83%	0.21
	Barefoot vs. Cut-out CFO	2.844	*p* = 0.046	83–100%	0.57
	AFSE CFO vs. Cut-out CFO	2.760	*p* < 0.001	0–100%	0.33
Ankle transverse	-	-	-	-	
Midfoot transverse	-	-	-	-	

**Table 3:** First metatarsophalangeal joint moment.

		Critical Threshold	Probability Value (*p*)	Percentage of the Stance Phase	Cohen’s d
first MTPJ X Axis	-	-	-	-	
first MTPJ Y Axis	-	-	-	-	
first MTPJ Z Axis	Barefoot vs. AFSE CFO	4.047	*p* < 0.001	20–83%	0.31
	Barefoot vs. AFSE CFO	4.047	*p* = 0.054	96–98%	0.19
	Barefoot vs. Cut-out CFO	-	-	-	
	AFSE CFO and Cut-out CFO	4.138	*p* < 0.001	61.81%	0.15

## Data Availability

The data are unavailable due to privacy or ethical restrictions; a statement is still required.

## References

[1] Hall C., Nester C.J. (2004). Sagittal Plane Compensations for Artificially Induced Limitation of the First Metatarsophalangeal Joint. J. Am. Podiatr. Med. Assoc..

[2] Canseco K., Long J., Marks R., Khazzam M., Harris G. (2008). Quantitative characterization of gait kinematics in patients with hallux rigidus using the Milwaukee foot model. J. Orthop. Res..

[3] Hernandez Gombau V. (2020). Ánalisis de Las Variables Baropodométricas En El Hallux Limitus Mediante Su Tratamiento Ortopodológico. Ph.D. Thesis.

[4] Dananberg H.J. (1993). Gait style as an etiology to chronic postural pain Part, I.I. Postural compensatory process. J. Am. Podiatr. Med. Assoc..

[5] Van Gheluwe B., Dananberg H.J., Hagman F., Vanstaen K. (2006). Effects of hallux limitus on plantar pressure and foot kinematics during walking. J. Am. Podiatr. Med. Assoc..

[6] Kirby Kevin A. (2000). Normal and Abnormal function of the foot. J. Am. Podiatr. Med. Assoc..

[7] Teixeira R.R. (2008). Biomecánica del primer radio. Deformidad en flexión plantar. Caso clínico. Rev. Int. Cienc. Podol..

[8] Dananberg Howard J. (1986). Functional hallux limitus and its relationship to gait efficiency. J. Am. Podiatr. Med. Assoc..

[9] Blázquez R., Lázaro J.L., García E., Gonzalez M.L. (2010). Relación del Índice Postural del Pie Con el Hallux Limitus Funcional.

[10] Fung J., Sherman A., Stachura S., Eckles R., Doucette J., Chusid E. (2020). Nonoperative Management of Hallux Limitus Using a Novel Forefoot Orthosis. J. Foot Ankle Surg..

[11] Clough James G. (2005). Functional hallux limitus and lesser-metatarsal overload. J. Am. Podiatr. Assoc..

[12] Durrant B. (2009). Functional hallux limitus. J. Am. Podiatr. Med Assoc..

[13] Munuera Martínez P. (2008). El Primer Radio. Biomecánica y Ortopodología.

[14] Munuera Martínez P.V. (2006). Factores Morfológicos en la Etiología del Hallux Limitus y el Hallux Abductus Valgus.

[15] Vanore J.V., Christensen J.C., Kravitz S.R., Schuberth J.M., Thomas J.L., Weil L.S., Zlotoff H.J., Mendicino R.W., Couture S.D. (2003). Diagnosis and Treatment of First Metatarsophalangeal Joint Disorders. Section 2: Hallux Rigidus Clinical Practice Guideline First Metatarsophalangeal Joint Disorders Panel. J. Foot Ankle Surg..

[16] Caravelli S., Mosca M., Massimi S., Pungetti C., Russo A., Fuiano M., Catanese G., Zaffagnini S. (2018). A comprehensive and narrative review of historical aspects and management of low-grade hallux rigidus: Conservative and surgical possibilities. Musculoskelet. Surg..

[17] Camasta C.A. (1996). Hallux limitus and hallux rigidus. Clinical examination, radiographic findings, and natural history. Clin. Podiatr. Med. Surg..

[18] Shamus J., Shamus E., Gugel R.N., Brucker B.S., Skaruppa C. (2004). The Effect of Sesamoid Mobilization, Flexor Hallucis Strengthening, and Gait Training on Reducing Pain and Restoring Function in Individuals with Hallux Limitus: A Clinical Trial. J. Orthop. Sports Phys. Ther..

[19] Colò G., Fusini F., Samaila E.M., Rava A., Felli L., Alessio-Mazzola M., Magnan B. (2020). The efficacy of shoe modifications and foot orthoses in treating patients with hallux rigidus: A comprehensive review of literature. Acta Biomed..

[20] Smith C., Spooner S.K., Fletton J.A. (2004). The Effect of 5-Degree Valgus and Varus Rearfoot Wedging on Peak Hallux Dorsiflexion during Gait. J. Am. Podiatr. Med. Assoc..

[21] Munuera P.V., Domínguez G., Palomo I.C., Lafuente G. (2006). Effects of Rearfoot-Controlling Orthotic Treatment on Dorsiflexion of the Hallux in Feet with Abnormal Subtalar Pronation. J. Am. Podiatr. Med. Assoc..

[22] Scherer P.R., Sanders J., Eldredge D.E., Duffy S.J., Lee R.Y. (2006). Effect of Functional Foot Orthoses on First Metatarsophalangeal Joint Dorsiflexion in Stance and Gait. J. Am. Podiatr. Med. Assoc..

[23] Kerry K. (2003). Rambarran. Effectiveness of the Kinetic Wedge Foot Orthosis Modifitcation to Improve Gait Posture.

[24] Menz H.B., Auhl M., Tan J.M., Levinger P., Roddy E., Munteanu S.E. (2016). Biomechanical Effects of Prefabricated Foot Orthoses and Rocker-Sole Footwear in Individuals with First Metatarsophalangeal Joint Osteoarthritis. Arthritis Care Res..

[25] Welsh B.J., Redmond A.C., Chockalingam N., Keenan A.M. (2010). A Case-Series Study to Explore the Efficacy of Foot Orthoses in Treating First Metatarsophalangeal Joint Pain. J. Foot Ankle Res..

[26] De Bengoa Vallejo R.B., Gómez R.S., Iglesias M.E.L. (2016). Clinical improvement in functional hallux limitus using a cut-out orthosis. Prosthet. Orthot. Int..

[27] Bennell K., Talbot R., Wajswelner H., Techovanich W., Kelly D., Hall A. (1998). Intra-rater and inter-rater reliability of a weight-bearing lunge measure of ankle dorsiflexion. Aust. J. Physiother..

[28] Gatt A., Mifsud T., Chockalingam N. (2014). Severity of pronation and classification of first metatarsophalangeal joint dorsiflexion increases the validity of the Hubscher Manoeuvre for the diagnosis of functional hallux limitus. Foot.

[29] Mohamed O., Cerny K., Jones W., Burnfield J.M. (2005). The effect of terrain on foot pressures during walking. Foot Ankle Int..

[30] Bruening D.A., Cooney K.M., Buczek F.L. (2012). Analysis of a kinetic multi-segment foot model. Part I: Model repeatability and kinematic validity. Gait Posture.

[31] Bruening D.A., Cooney K.M., Buczek F.L. (2012). Analysis of a kinetic multi-segment foot model part II: Kinetics and clinical implications. Gait Posture.

[32] Sanchis-Sales E., Sancho-Bru J.L., Roda-Sales A., Pascual-Huerta J. (2016). Dynamic Flexion Stiffness of Foot Joints during Walking. J. Am. Podiatr. Med. Assoc..

[33] Gordillo-Fernández L.M., Ortiz-Romero M., Valero-Salas J., Salcini-Macías J.L., Benhamu-Benhamu S., García-De-La-Peña R., Cervera-Marin J.A. (2016). Effect by custom-made foot orthoses with added support under the first metatarso-phalangeal joint in hallux limitus patients. Prosthet. Orthot. Int..

[34] Sánchez-Gómez R., López-Alcorocho J.M., Núñez-Fernández A., Fernández M.L.G., Martínez-Sebastián C., Ortuño-Soriano I., Zaragoza-García I. (2023). Morton’s Extension on Hallux Rigidus Pathology. Prosthesis.

[35] Hajizadeh M., Desmyttere G., Ménard A.L., Bleau J., Begon M. (2022). Understanding the role of foot biomechanics on regional foot orthosis deformation in flatfoot individuals during walking. Gait Posture.

